# “I decide for myself. I’m like the boss of myself. I can cook when I want.” Prisoners’ experiences with a self-catering food system in a Norwegian low-security prison

**DOI:** 10.1186/s40352-026-00426-z

**Published:** 2026-06-12

**Authors:** Astrid Kolderup Hervik, Stein Egil Kolderup Hervik

**Affiliations:** Faculty of Humanities, Sports and Educational Sciences, University of South-Eastern Norway, Horten, Norway

**Keywords:** Self-catering, Prison, Health promotion, Agency, Lay perspectives, Prisoners

## Abstract

**Background:**

Food is a central part of everyday life in prison, carrying meanings and values that shape identity and social relations and may have important implications for health and well-being. Previous research suggests that self-catering systems, whereby prisoners plan, shop for, and prepare their own meals, may increase opportunities for agency, participation, and health promotion. Despite growing interest in prison food systems, little empirical research has explored how prisoners themselves experience such self-catering arrangements. This study explores prisoners’ experiences with a self-catering food system in a Norwegian low-security prison, with particular attention paid to its potential for promoting health and well-being.

**Method:**

The study draws on 20 individual semi-structured interviews with male prisoners aged between 20 and 70, representing diverse ethnicities and social backgrounds. The interviews were analysed using Braun and Clarke’s six-phase thematic analysis.

**Results:**

Four themes were developed through the analysis, reflecting prisoners’ experiences of the self-catering system: food-related agency, social skills and relations, food-related skills, and support and guidance. Prisoners generally described self-catering as a meaningful increase in agency, offering control over day-to-day food practices, dietary preferences, and identity work. Socially, the self-catering system facilitated social relationships through co-operation, mutual support, and shared routines that fostered experiences of normality, emotional well-being, and social cohesion. Collaborative food groups often supported skill development, healthier eating and cost-sharing. However, experiences varied widely. Prisoners who lacked supportive social networks, sufficient financial resources, or food-related skills and competencies faced significant barriers. Many described a need for support and guidance, such as cooking courses or mentoring.

**Conclusion:**

The study illustrates how food-related practices enabled by a self-catering system may function as meaningful aspects of everyday life in prison. When adequately supported, self-catering systems can serve as one way to promote health within prison settings. However, limited resources to take advantage of food-related agency, including aspects related to food literacy and lack of social support, emerged as key barriers to realising the health-promoting potential. To more fully realise such potential, self-catering systems require attention to be paid to inequalities, support needs, and structural conditions.

## Background

Prisoners typically experience poorer health compared to the general population, with a higher prevalence of lifestyle behaviours that increase the risk of health problems (e.g. Baybutt & Chemlal, 2016; Baybutt et al., 2019; Bukten et al., 2016; Goldblatt et al., 2023). Systematic reviews show elevated risk of non-communicable diseases (NCDs) (Munday et al., 2018; Solares et al., 2020), alongside increased prevalence of infectious diseases, such as hepatitis C and HIV among prison populations (e.g. Baybutt & Chemlal, 2016; Hajarizadeh et al., 2024). Studies further demonstrate higher prevalence of mental health disorders and substance use problems among prisoners, both internationally (e.g. Baranyi, 2022; Di Lorito et al., 2018) and in Norway (Bukten et al., 2016, 2024; Tverborgvik et al., 2025). Healthy dietary patterns contribute to better health outcomes and can, for example, reduce the risk of NCDs, whereas unhealthy diets increase this risk (Willett et al., 2019). These effects are closely linked to other behavioural risk factors, as multiple health behaviours collectively shape health outcomes (Schroeder, 2007). In prison populations, where multiple risk factors may be present, nutrition and food-related behaviours represent a potential entry point for health promotion and disease prevention (Condon et al., 2008; Smoyer & Minke, 2015).

Health literacy encompasses the social and cognitive skills that enable individuals to access, understand, and use information to promote and maintain health and achieve empowerment (Nutbeam, 2000). Research indicates that prisoners may have lower levels of health literacy, reflecting broader social and educational inequalities (Mehay et al., 2021). Building on this broader conceptualisation, food literacy has emerged as a related framework that focuses more specifically on food (Hernandez et al., 2021). Although definitions vary (Hernandez et al., 2021), food literacy is commonly understood as encompassing interrelated knowledge, skills, and practices related to planning, selecting, preparing, and eating food in ways that support appropriate and nutritionally adequate food choices in the context of everyday life (Vidgen & Gallegos, 2014). Food literacy further encompasses the capacity to navigate food environments and adapt to changing circumstances over time, and it is shaped by the broader social and environmental context in which individuals live (Hernandez et al., 2021; Vidgen & Gallegos, 2014). In their outline of a Food Literacy Conceptual Model, Hernandez et al. (2021) emphasise that food literacy is not only shaped by social and environmental conditions, but also by broader economic, cultural, and political forces. At the core of this model are competencies of understanding, accessing, appraising and applying information related to food and health as central to food literacy. Previous research has identified a range of vulnerabilities and challenges related to food among prisoners, including limited knowledge, lack of cooking skills, and contextual and individual barriers to making healthy food choices (Condon et al., 2008; Hervik & Hervik, 2025; Minke, 2014). These challenges relate to key components of food literacy and may suggest varying and, for some, limited levels of food literacy among prisoners.

Prisons provide a unique opportunity to reach individuals who may otherwise be difficult to reach with health promotion measures and are therefore considered important settings for health promotion (Baybutt et al., 2014). Crawford (2006) argues that health should be understood within a broader social and cultural context, promoting a holistic approach that considers lived experiences and socio-cultural meanings. From this perspective, health is not merely a biological state or medical outcome, but a meaningful social practice embedded in everyday life. Health is thus shaped through social practices and the contexts in which they occur (Crawford, 2006). Consequently, the development of an understanding of social practices in prisons is crucial for promoting health in these contexts. Food is one such social practice, carrying different symbolic, social, rational and emotional meanings and values central to everyday life in prison (Hervik & Hervik, 2025). Understanding these comprehensive meanings and values can provide valuable insights for those designing prison policies and health promotion strategies (Hervik & Hervik, 2025). Hence, food and eating are central elements of prison life, not only in terms of nutritional intake and biological aspects of health, but also because food and eating are embedded in everyday practices that shape how health and well-being are experienced and managed.

Food and eating may also be understood in relation to identity. Food practices communicate “who we are” in different ways, symbolising personal, social, and cultural identity; through food, identity is constructed, reconstructed, and negotiated as a means of expressing belonging and distinction (Monterrosa et al., 2020). Enabling everyday routines and roles such as food preparation and shared meals can contribute to identity work and to the fostering of a sense of control among prisoners, factors which can be important for prisoners’ health and well-being (Hervik & Hervik, 2025; Vanhouche, 2022). Identity has been understood as fluid and continuously constructed through desires, choices, behaviours, and social relationships (Elliott, 2020; Giddens, 1991; Goffman, 1969). In restrictive environments such as prisons, maintaining a sense of self becomes particularly important (Vanhouche, 2022). Vanhouche (2022) and Hervik and Hervik (2025) highlight how prisoners engage in identity work through food practices to sustain a coherent sense of self under such constraints, with Hervik and Hervik (2025) further indicating that such processes may also have implications for prisoners’ well-being and overall health.

These understandings align with the principle of normalisation, which guides the Norwegian correctional system and holds that everyday life in prison should resemble life in the wider community as closely as possible (Ministry of Justice and Public Security, 2021), an ideal also articulated in the *European Prison Rules*, which state that “life in prison shall approximate as closely as possible the positive aspects of life in the community” (Council of Europe, 2020). Normalisation thus reflects a broader ideal that seeks to minimise the gap between prison and society, support prisoners’ well-being, and prepare prisoners for life after release. In the Norwegian context, this principle extends beyond formal rights and institutional routines to encompass the ordinary, practical dimensions of everyday life. A self-catering food system, in which the prisoners themselves plan, shop for, and prepare their own meals, is presented as an example of prison normalisation (Engbo, 2017). In many Norwegian prisons, prisoners are expected to take some responsibility for tasks such as budgeting, food shopping, cooking, and maintaining household routines, and such everyday practices offer a lens through which to examine how food-related activities may shape experiences of agency, sociality, and rehabilitation (cf. Towns & Ricciardelli, 2024). The way in which the principle of normalisation is implemented may play an important role in shaping prisoners’ well-being, as well as their opportunities for personal development and successful reintegration upon release. This article addresses the broader issue of how prisoners experience self-catering systems within the prison environment.

### Previous research on self-catering systems in prison settings

Smoyer and Minke (2015) argue that food systems in correctional settings can play an important role in shaping both health and behavioural outcomes. They highlight that food systems may influence eating behaviours, which in turn can affect physical health - including through the prevention of NCDs when supporting healthier dietary practices - as well as mental, social, and cultural aspects of health. Research has highlighted that restrictions in food-related agency may have negative effects on prisoners’ health and well-being (Godderis 2006a, b; Minke 2014; Smith 2002; Ugelvik 2011; Vanhouche 2022). Lack of control over food decisions or experiences with poor food quality can turn food into a symbol of punishment and may reflect broader constraints on identity and routine (Johnson et al., 2024; Vanhouche, 2022). In contrast, systems that allow for higher degrees of food-related agency – such as self-catering arrangements – have been associated with several individual and social benefits (Hervik & Hervik, 2025). Similarly, Groeneveld et al. (2025) emphasise that prisoners appreciate opportunities for agency in food-related practices, and that the normalisation of carceral food spaces plays a meaningful role in their everyday prison experience.

However, prisoners constitute a heterogeneous group, and this variation is reflected in differences in areas such as what and how they eat, their level of nutritional knowledge, and their skills in meal planning and cooking (Hervik & Hervik, 2025). This indicates a need to consider that the potential benefits of self-catering may be unevenly distributed, as some individuals may face constraints related to limited food literacy.

Research from European and Nordic contexts shows a consistent pattern in how prisoners experience self-catering systems. A European study by Vanhouche (2022) demonstrated that self-catering systems gave prisoners agency and opportunities to organise meal planning, shopping, cooking, and cleaning together in food groups. Such co-operation and social bonding promoted inclusion for those with limited resources, facilitated cost-saving, helped reduce isolation, and improved emotional well-being. These experiences were in turn shaped by material conditions, such as access to equipment, storage, and kitchen cleanliness. Furthermore, the self-catering system fostered a sense of independence and was important for prisoners to (re)construct and express their identity (Vanhouche, 2022). The shared experience of cooking and eating including cross-cultural food practices, could also help break down otherwise rigid social barriers (Vanhouche, 2022). However, structural limitations and inequalities persisted despite the system’s benefits. Food-related agency was, for example, closely tied to prisoners’ economic status, with some prisoners viewing high food prices as a direct barrier to independence (Vanhouche, 2022).

Similar findings appear in Danish contexts. In a study of incarcerated women, Minke and Smoyer (2017) found that while self-catering arrangements increased agency and aligned daily life more closely with life outside prison, this potential was often constrained by structural factors such as high prices, limited shop selection, and chaotic or unhygienic kitchen environments. These structural barriers both restricted healthy food choices and highlighted social inequalities among prisoners (Minke & Smoyer, 2017). Among male prisoners, Minke (2014) likewise found that the self-catering system was preferred over institutional catering, as it allowed greater control over food intake and supported cultural expression, health awareness, and the (re)construction and expression of non-criminal identities. Co-operative food groups were common, often formed for economic reasons, and functioned as important arenas for building social capital, with roles and relationships often extending beyond food-related matters. As in other studies, however, high prices and limited product availability remained persistent sources of dissatisfaction.

The health-promoting potential of food-related agency has been highlighted in larger syntheses of prison food research. In a systematic review, Johnson et al. (2024) found that greater food-related agency was associated with higher prisoner satisfaction and suggested that empowering prisoners to participate in food preparation holds potential for promoting health in prison settings through increased opportunities for control and engagement in everyday food practices. At the same time, the authors observed that increased responsibility for meals requires food skills that not all prisoners possess.

Similarly, in a meta-ethnography of 17 studies from ten countries, Woods-Brown et al. (2023) found that allowing prisoners to cook and share meals may contribute to improved health and well-being through practical, social and mental processes. Shared cooking and eating activities were found to foster prisoner interaction, strengthen supportive relationships among prisoners, and mutual prisoner influence, as well as contribute to the development of life skills and to play an important role in prisoners’ identity work. Consequently, Woods-Brown et al. (2023) emphasise that systems with some degree of prisoner agency can empower prisoners, promote health, and support prisoner reintegration.

Although self-catering systems may offer an opportunity to exercise agency and provide a sense of normality, the meaning of “normality” is not universal. As Engbo (2017) points out, what is perceived as normal depends on a prisoner’s life history and social background. Some may associate normality with preparing their own meals, while others may be more accustomed to having a partner preparing their food or relying on takeaways. Similarly, Minke and Smoyer (2017) argue that the assumption that access to a kitchen in itself will promote healthy eating overlooks the diversity and vulnerability of the incarcerated population. Taken together, prior research illustrates the multifaceted roles of food in self-catering systems in prison settings, and points to both opportunities and challenges related to agency, social relationships, and everyday food practices.

### Theoretical framework

The World Health Organization (WHO) describes health promotion in the Ottawa Charter (1986) as a process that enables individuals and groups to improve and gain greater control over their health. This process involves key elements such as the dissemination of information, health education, and the development of life skills alongside structural and environmental measures. The Ottawa Charter highlights that health is generated within the context of everyday life, where people make decisions and take actions to care for themselves and others (World Health Organization, 1984). This view supports a settings-based approach to health promotion, which recognises environments such as schools, workplaces, and prisons as important arenas for shaping health outcomes through institutional structures and social dynamics (Green et al., 2019; Kickbusch, 1996; Poland et al., 2000).

A core concept within health promotion is empowerment, which refers to the “process through which people gain greater control over decisions and actions affecting their health” (Nutbeam & Muscat, 2021). According to Kayser et al. (2019), empowerment operates on three interrelated levels: the micro-level involves individual needs and experiences, the meso-level concerns institutional structures and values, and the macro-level reflects political and societal systems. Health promotion processes are particularly challenging in prisons, since prisons are contexts that present substantial barriers to promoting health and well-being (Condon et al., 2008; Green et al., 2019; Whitehead, 2006; Woodall, 2020; Woodall, Dixey et al., 2014). This is largely because health promotion is grounded in agency and empowerment (Green et al., 2019), while prison environments are structured to limit agency (Woodall, 2020; Woodall, Dixey et al., 2014). However, the degree of agency afforded to prisoners in a prison setting varies across prison systems worldwide.

In the Norwegian context, these institutional barriers are partly mitigated by the principle of normalisation (Van de Rijt et al., 2023). Comparative research indicates that prisoners in Norway experience higher levels of autonomy than in some other prison systems. For example, while 62% of prisoners in England and Wales feel they have little control over their day-to-day lives, the corresponding figure in Norway is 35% (Crewe et al., 2022). This difference may be related to the relatively extensive use of low-security prisons in Norway. Mjåland et al. (2023) found that approximately 50% of the Norwegian prison population spent time in low-security settings in 2019, compared to around 5% in England and Wales. At the same time, it is important not to romanticise the Norwegian penal system. Despite being more humane in relative terms, prisons in Norway remain punitive spaces in which prisoners continue to report considerable pain, frustration, and a diminished sense of agency (Crewe et al., 2022; Shammas, 2014).

At the same time, prison settings may hold potential for empowerment-focused health promotion measures. When prisoners are given opportunities for agency, such as through involvement in food-related decisions and practices, this may contribute to greater well-being (Hammond et al., 2024; Hervik et al., 2025; Johnson et al., 2024; Woodall, 2020). According to Woodall et al. (2014a, b), the promotion of health in prison contexts has traditionally been focused on medical models that emphasise personal responsibility and behavioural change. However, integrating the perspectives and lived experiences of lay people such as prisoners, can foster a more holistic and context-sensitive approach to health, potentially challenging dominant paradigms (Hervik, 2016; Hervik & Skille, 2021; Robertson, 2006).

While previous studies have shed light on various dimensions of prison food systems, there remains a limited body of qualitative research that centres on food from the perspective of prisoners themselves (Condon et al., 2008; Johnson et al., 2024; Woods-Brown et al., 2023). Additionally, Wilson (2023) contends that even if food has long been used as a medium for social change and challenges to power structures, this lens has not been fully utilised within prison contexts. She therefore calls for a more critical examination of how food systems within carceral environments could become tools for empowerment, relationship-building, and systemic reform.

Prison food systems may take different forms, including institutionally provided meals, canteen-based provision, and opportunities for prisoners to prepare food themselves (Smoyer & Minke, 2015). The setting in this study represents the latter context, making it possible to explore how prisoners experience a self-catering system. More specifically, the study explores how prisoners experience a self-catering food system within a Norwegian low-security prison context, with particular attention to its potential for promoting health and well-being.

## Materials and methods

### Context

Forest Prison (pseudonym) is a large Norwegian low-security men’s prison with a capacity of 125 prisoners. The prison population was heterogeneous in terms of age, sentence length and nationality. The facility primarily houses prisoners transferred from high-security prisons as part of a gradual transition towards release, and thus functions as a preparatory setting for reintegration into society. To preserve anonymity in a relatively small population, detailed demographic distributions are not reported. The prison is organised like a small village, with residential houses, a grocery store, and various shared amenities. While some prisoners live alone, most reside in single rooms within shared houses.

At the time of data collection, the prison had a self-catering food system, which had been relatively recently introduced. Previously, dinners had been provided through an institutional catering system, with meals served in the prison dining hall, but this arrangement was discontinued. For a brief period, prisoners were provided with ready-made meals to heat in their housing units. Widespread dissatisfaction with this solution led to the introduction of the self-catering system. Under the self-catering system, prisoners received a daily allowance of NOK 118 (approximately €11), intended to cover all meals. In addition, all prisoners were required to work and received a daily wage ranging from NOK 70 to NOK 100 (approximately €7–€10), depending on the type of work performed. Prisoners could also supplement their income with private funds, if available, or receive financial support from family members outside the prison, although access to such resources varied. No regular meals were provided by the prison, and prisoners were responsible for planning, budgeting, shopping for, and preparing their own food. Food had to be purchased from the prison grocery store. Access to food was therefore shaped by the availability, quality, and pricing of products in the store. Some prisoners established food groups within their residences, collaborating on various aspects of food-related processes and tasks.

### Method

This study adopted a qualitative design, aiming to explore prisoners’ experiences of the self-catering system. Individual semi-structured interviews were chosen as the method of data collection, as they are well-suited to capturing personal experiences, and allow for the discussion of personal topics in a confidential setting.

The study was developed and conducted in close collaboration with prison management, staff, and prisoners. Two resource groups, one consisting of two prisoners and the second consisting of seven members of the staff, both contributed to the development of the interview guide and recruitment strategy. All prisoners were informed about the study during a “head count”, a routine procedure in which all prisoners are gathered and counted, which led to the immediate recruitment of a substantial portion of the sample. To enhance sample diversity, a prisoner who had been involved in the planning phase acted as a gatekeeper. Holding a central social position among prisoners, the gatekeeper informed peers, and encouraged participation across age groups, and cultural and social backgrounds. This purposive sampling was employed to ensure variation in ethnicity, age, and social background (cf. Patton, 2015).

In the spring of 2022, individual interviews were conducted with 20 prisoners aged between 20 and 70. The participants represented diversity in terms of nationality, ethnicity, and both social and criminal backgrounds. Three interviewers (including both authors) conducted the interviews individually. To ensure a consistent approach, the first interviews were conducted jointly and were followed by debriefs and adjustments. Interviews were held in peripheral prison buildings to promote open dialogue in a setting where participants felt less observed. The interviews lasted between 28 and 135 min and were digitally recorded and transcribed verbatim.

### Analysis

Both authors were actively involved in the analytical process, which was informed by a phenomenological approach aimed at understanding the prisoners’ own experiences. The analysis followed the six-phase approach to thematic analysis by Braun and Clarke (2006). In phase one, both authors familiarised themselves with the data by reading all the transcripts and listening to the audio recordings. Author 1 then proceeded to the second and third phases, generating initial codes and identifying potential themes, while maintaining regular dialogue with Author 2 to ensure reflexivity and analytical depth. In phases four and five, the authors collaborated closely to review, refine, and define the themes, working to ensure that the emerging themes accurately reflected the participants’ expressed experiences. During this process, particular attention was paid to capturing the essence of each theme and its relevance across the interviews. In phase six, both authors jointly completed the final analysis and contributed actively to the writing. Preliminary analytical insights were shared with prisoners and staff at the prison. Their feedback confirmed that the findings resonated with their experiences, supporting the credibility of the identified themes.

## Results

### The path to a self-catering system in Forest Prison

Just prior to the introduction of the self-catering system at Forest Prison, dinners were served in the prison dining hall. This contextual detail is important, as prisoners’ experiences with this earlier system shaped their perceptions of the self-catering system, with frequent comparisons drawn between the two. Some prisoners expressed satisfaction or partial satisfaction with having dinners served, particularly those with limited cooking skills or those who conceptualised food primarily as sustenance. However, many voiced extensive criticisms concerning the quality of the food, the ingredients used, how ingredients were handled, the overall management of the kitchen, including the culinary competence of the staff, and the overall food quality. Sebastian recounted his dismay regarding the food served in the prison dining hall: “I was quite shocked when I arrived here (…) There was nearly a complete absence of vegetables in the dinners.” Negative food experiences in the former food system were also described as leading to conflicts. Adrian, for instance, suspected financial misappropriation: he believed that the prison was embezzling money allocated for meals, which reduced his sense of well-being in the prison.

Some weeks before the interviews were conducted, the prison dining hall was scheduled for renovation, and ready-made meals were introduced as a temporary replacement for dinners served. These meals, which prisoners were required to heat in their houses, quickly became a source of widespread dissatisfaction. They were generally perceived as both unappetising and insufficient in quantity. Erik described the food as “a joke”, while William remarked, “Even the staff turn up their noses at the food.” Ron similarly criticised the meals, stating, “It tastes like plastic. It tastes like nothing” and noted that this negatively affected his mental well-being.

This discontent culminated in collective action. Dissatisfaction with the ready-made meals galvanised organised protest, advocating for a self-catering model wherein prisoners would receive financial allowances to purchase and prepare their own food. The prisoners organised a petition that reportedly accumulated around 110 signatures, which ultimately contributed to a change in the food system and the introduction of self-catering. Ayo emphasised the important role of a newly appointed manager in achieving this goal: “We succeeded. He was willing to give us money, and everyone was happy”.

The introduction of a self-catering system was a significant event for many prisoners, who spoke about it and the manager responsible for its implementation, with great appreciation. William simply stated, “He got the picture.” Noah also praised both the new food system and the manager, characterising him as humble and referring to him as a great leader and a very good man – a manager they could trust.

### Prisoners’ experiences with the self-catering system

In the following, the findings related to prisoners’ experiences with the self-catering system are presented. Prisoners described how this system was related to their physical, mental, and social health and well-being in several ways. Four themes emerged from their accounts: Food-related agency; Social skills and relations; Food-related skills; and Support and guidance.

#### Food-related agency

The findings indicate that food-related agency was of great importance to prisoners. Issues related to food emerged as one of the relatively few areas in which prisoners could exercise individual choice and decision-making, and food thus came to represent their broader sense of agency within the prison environment. Accordingly, the introduction of the self-catering system was experienced as an expansion of opportunities to make their own choices and decisions, which many prisoners described as important.

Robert asserted, “They [the prison management] shouldn’t decide what I eat”, while Roger emphasised the value of agency, stating, “I decide for myself. I’m like the boss of myself. I can cook when I want.” Many prisoners highlighted the opportunity the system afforded to purchase and prepare meals of their own choice. Jonas noted that it gave them the opportunity to enjoy what they personally considered good food. Prisoners described diverse eating routines, and the self-catering system enabled them to eat in ways that suited their individual needs. This provided a meaningful opportunity to exercise control over a fundamental aspect of everyday life, which was described as important for their well-being. This was particularly relevant for prisoners engaged in regular physical training, for whom meal timing and portion control were essential. Martin, one of these prisoners, described this experience of increased agency:

I want to eat when I want to eat [laughs] and when I’m hungry. Sometimes I eat a bit more, other times a bit less. I like to be a bit free, in that sense. I don’t feel so trapped.

Food-related agency was experienced not only as a practical benefit but also important to the expression of individuality. As food was one of the relatively few areas where prisoners could exercise control, it became an important way for them to express who they were. In this way, the self-catering system played an important role in enabling prisoners to (re)construct and express different facets of identity, shaped by factors such as culture and ethnicity, masculinity, and health consciousness. Ayo, for example, who came from an African country, emphasised that preparing African food allowed him to connect with his family and feel a sense of home. He was able to prepare traditional dishes under the self-catering system, which he valued highly, especially in contrast to his earlier experiences in closed prisons, which he described as “a hell.” Moreover, some prisoners described distancing themselves from previous behaviours and fostering a sense of self aligned with positive personal development, such as acquiring cooking skills or adopting a healthier diet.

On the other hand, when prisoners perceived a reduction in agency within the self-catering system, it was described in highly negative terms. Such experiences were often linked to the prison store. Several participants criticised the quality of its products, the limited selection, and high prices – all factors that curtailed their food-related agency. These limitations were also described as barriers to making healthier food choices. Erik, for example, criticised the poor quality of the store’s vegetables, describing it as “a tragedy,” with examples such as rotten tomatoes and soft cucumbers. He elaborated: “…the selection they have for vegetables is below all criticism. And that’s exactly why, instead of buying myself a good salad, I end up getting sausages and hot dog buns instead…”.

There were economic inequalities among prisoners; some experienced having sufficient financial resources and could buy and eat what they wanted, while others struggled to afford sufficient or nutritious food. Philip expressed frustration with pricing, especially in light of prisoners’ limited incomes:

You must pay high prices for absolutely everything. And there’s a limited selection, so you can’t get far with 59 kroner at the price level here. If I go to Rema [Norwegian low-cost grocery store], I can buy the same items for half the price, but okay, at least we have this option here.

Ayo also raised concerns about high prices, noting that this also sometimes led to suspicion among prisoners. He explained:

Everyone says, ‘They’re stealing money from us.’ It is extremely expensive compared to outside. It’s very expensive. That raises questions. People start saying, ‘Oh, they’re taking money from us? (…) and if you’re not smart, you think, ‘Yeah, that’s true, they’re stealing from us.’ But they’re not.

This range of concerns illustrates how limited food selection, high costs, and poor product quality in the prison store reduced prisoners’ experiences of agency within the self-catering system, experienced as reducing their well-being.

#### Social skills and relations

The self-catering system was experienced by many prisoners as having important social value, shaping everyday interactions, relationships, and ways of supporting one another. Through shared food practices, prisoners described developing social relationships and skills such as co-operation and consideration for others, while also creating routines, rituals, and moments of normality that supported their sense of community, care, and social well-being. The value of shared meals and their social dimension was widely acknowledged among prisoners. Ayo remarked, “When I eat together with others, the food tastes better.” Ron similarly explained:

It’s not the waffle that’s the most important. After all, all that matters is the people sitting around the table. It makes everything more pleasant, and it does something to you. Every day becomes a bit lighter… it’s the whole setting. You know what it’s called? Normality.

Food-related social interactions were described as important for prisoners’ mental well-being. Ron explained how a shared meal improved his mood on a difficult day:

On Monday, I was having a tough day. I felt a strong yearning for home, missing my family terribly … But then, someone knocked on my door, asking if I wanted to join them outside. And there they were barbecuing. It wasn’t anything extravagant – just a simple salad and a neck chop. But the experience of sharing that meal and sitting around the table in nice weather, with good company and good food, brought a sense of order back into my world.

In some houses, shared routines such as weekend barbecues, taco nights, and birthday celebrations helped recreate a sense of life outside prison and fostered a sense of normality. These routines created something to look forward to and gave structure to day-to-day life. Tom explained, “It’s almost exactly like outside, living in a community. We meet in the evenings, have dinner together around seven, and whoever has the most time cooks.” Ayo similarly highlighted the significance of social happenings with food, describing how watching the Champions League final with snacks and soda “felt much more like home.” More informal gatherings, such as baking cakes or making waffles, were also described as moments of warmth and joy by many prisoners. By establishing shared routines and planning events, prisoners created opportunities for social bonding and agency and formed small, meaningful rituals that were described as creating a sense of normality and contributing to their well-being. For some prisoners who had lacked structure prior to incarceration, these routines also provided a newfound sense of stability.

Planning, preparing, and sharing meals fostered social relationships, co-operation and mutual support among many prisoners. This was particularly evident among those who formed food groups, as they regularly shared meals and collaborated on food-related tasks. Participation in food groups varied across housing units, but where collaboration was more established, working together on meals facilitated the development of important social skills, such as negotiation, compromise, and care for others. Beyond functioning as a social skill developed through collaboration, care was also expressed through concrete food-related practices. Food served as a means of showing care and concern for others, as cooking and sharing meals were often regarded as meaningful expressions of kindness. Tom described living in a house where people frequently visited and shared meals, and where he actively sought to ensure that everyone felt included in the social community. Similarly, Philip described baking pizza and preparing meals for prisoners who were locked in during the COVID-19 pandemic, which these prisoners described as “the greatest care they had received in prison.” Ron likewise emphasised the significance of small, meal-related gestures: “If someone is staring down at their oatmeal and is quiet, you ask, ‘Hey, are you okay?’ and they say, ‘Yes, thanks for asking.’ It’s that little bit of care. People care about each other here.”

Food practices were also tied to cultural sharing and mutual acceptance. Ayo, who often cooked African dishes, initially worried that his housemates might find the smells and time-consuming cooking methods intrusive. However, once they tasted his food, they welcomed it enthusiastically. “You need to make more. It was good.” Ayo expressed joy in this acceptance, saying, “You could eat all I make at once; it’s no trouble for me. It just makes me happy.”

Although communal eating was central to the social dimension of the self-catering system, not all prisoners participated in food groups. Some were socially isolated or excluded from prison communities and therefore did not experience the social opportunities the system could offer. Others chose to cook alone, stating a need for privacy, diversity in needs among housemates, or discomfort with group settings. Jonas, for example, preferred shopping and cooking alone as a way to maintain personal space, which some prisoners highly valued. As Martin concisely put it, “I don’t like too much communal stuff.”

However, some prisoners who did not belong to food groups still expressed appreciation for the social potential of the self-catering system. Philip, for example, described occasionally preparing food together with others as a positive initiative for well-being: “Sometimes we try to make something together – some cakes or pastry from time to time – as a positive initiative for our well-being.” Similarly, Ayo, who usually preferred solitude due to his housemates’ large food intake, described finding joy in occasionally cooking for others: “I tell them, ‘Tomorrow you don’t need to cook. I’ll make the food,’ and they are very happy.” These accounts illustrate how food, within the self-catering system, could also serve as a means of contributing to social well-being among prisoners who were not part of food groups.

##### Social influence

In Forest Prison, social influence played a significant role in shaping prisoners’ eating habits and lifestyle choices. Through shared living spaces, co-operation, and common routines, prisoners influenced each other by encouraging healthier food choices, adhering to unwritten rules around food and nutrition, or showing respect and consideration through food-related behaviour. Kim described how another prisoner influenced his food choices, reluctantly accepting “fish dinner days” organised by a fellow prisoner despite his initial resistance. He remarked: “I don’t like giving him credit for it, but yes… he has a little bit of influence on me. Unfortunately, he actually does.” This illustrates how prisoners could shape each other’s behaviours, even when the influence is not entirely welcome.

Some prisoners assumed informal leadership roles, offering guidance and encouragement to others. Sebastian reflected on his own role in influencing his housemates: “I can speak in a humorous way that isn’t just dissing others. We have a couple of people who are up at night eating, and I try to do something about it without being too harsh on them.” He further highlighted how prisoners supported each other in making lifestyle changes: “I see some individuals are very good at helping each other with doing some [lifestyle] projects. We have someone who has been helping a roommate lose weight, and it’s easy to see that he has really made great progress”. These accounts show how informal social interaction can influence health-related behaviour in prison. The self-catering system thus created space for mutual influence and adaptation, shaping both individual and collective food and meal practices.

##### Social norms and status

Social status was also tied to food choices. In certain groups, prisoners who purchased and consumed healthy food earned higher status, while those who favoured junk food were associated with lower status. This suggested the existence of subtle yet powerful social norms around healthy eating and a culture of mutual accountability. These norms extended beyond the housing units, and into the grocery store. Tom described how food choices were monitored and commented on while shopping: “You can get teased if you’re standing in line with just chips and snacks, no vegetables, no chicken fillets, and no tuna cans (…) you can’t just have ready-made food. That’s not allowed.” Unwritten rules governed what was acceptable to buy, such as avoiding chips in the middle of the week, and those who deviated from these norms could face verbal correction. Even when interpersonal tensions existed, prisoners often adhered to shared group norms. Tom described how meal preparation required compromise:

Two wanted rice, but we are four against two, so we don’t make rice for just two people. (…) If they were alone, they would have made rice for maybe five people, and they would have eaten that rice, more than they need. But here, it becomes a balance … there’s no arguing.

Similarly, Erik explained how he expected to adapt to established food routines when moving into a new housing unit:

I spoke with the guys at the house I’m moving to, and there … they each contribute around 250 to 300 kroner, and then they buy food for the entire week. They take turns cooking, so I’ll have to adapt to that routine.

In some cases, food-related norms extended beyond food practices and structured everyday interactions among prisoners. One prisoner described living with someone he disliked but still adhering to household food rules: “Terrible paedophile, but NN (fellow prisoner) says I’m not allowed to bother him. And he made a soup yesterday that was awful (…) but I am not allowed to bother him.”

Taken together, the findings on social skills and relations suggest that the self-catering system shaped prisoners’ social lives in ways that extended beyond food, contributing to the development of social skills, relationships, and everyday routines associated with well-being. Social interactions within this context also influenced norms and practices related to food and eating among some prisoners.

#### Food-related skills

The self-catering system provided opportunities for prisoners to develop a range of food-related skills, including meal planning, cooking, maintaining kitchen hygiene, budgeting, and making healthier food choices. In houses with well-organised food groups, these skills were developed through structured routines for planning, shopping, cooking, and cleaning, contributing to skill acquisition and, for some prisoners, to healthier eating and more organised communal living.

Tom, who lived in a house with a highly functional food group, described how meals were planned collectively, with shopping typically taking place once a week. Cooking and cleaning responsibilities were shared through a rotational system: “We make everything together, we clean up after ourselves (…) you cook for three days and do the dishes.” He further stated, “It’s almost like being outside, really.” A similar routine was described by Sebastian, who emphasised the food group’s focus on nutritious meals: “We cook good dinners every day in our house and have fish twice a week.”

Many prisoners experienced skill development and learning within food groups. More experienced prisoners often assumed informal leadership roles, supporting others in developing skills related to meal preparation, nutrition, and budgeting. Tom described how older prisoners mentored younger ones in cooking and food planning, and how this knowledge-sharing fostered both the development of skills and social interaction. Sebastian likewise reflected on his own development, noting an emerging interest in cooking: “I have now started dreaming a bit about being able to cook some food. This has just come recently.”

Some prisoners reported eating more healthily and adopting healthier lifestyles under the self-catering system, particularly within well-functioning food groups. However, experiences varied, and some prisoners described their own or others’ diets as poor. Michael, for example, remarked that some would “make cake after cake after cake.” Prisoners outside food groups often identified several barriers to healthier eating, including the high cost of nutritious food, limited nutritional knowledge, and insufficient food-related skills such as meal planning and cooking.

This difference was also reflected in prisoners’ financial experiences. Prisoners in food groups often reported better financial outcomes due to collective shopping and meal planning. Sebastian emphasised that finances were not a constraint in his group, noting that they were able to plan meals without having to make trade-offs based on cost. This contrasted with the experiences of prisoners outside food groups, who more often described financial constraints and frustrations related to food expenses.

While food groups often generated positive outcomes, their functionality varied considerably. Some groups operated smoothly, with shared responsibility and co-operation, while others struggled with uneven participation or conflict. Adrian noted that although roles were assigned, some prisoners did not engage with their responsibilities. Others described tensions arising when certain individuals assumed dominant roles, directing or correcting others in ways that created frustration. Jimmy described how such dynamics could reduce well-being:

We have a house leader who is completely useless and who is so damn rude, so you have to scrub down the kitchen almost every time you cook. (…) He’s a nagging, yapping old woman, who stands there and hovers over you to find faults in what you’re doing, and it makes the whole thing very unpleasant.

In other cases, food groups were not arranged, or some prisoners chose not to join due to personal preferences. Some prisoners who cooked individually described shared kitchens as overcrowded and poorly equipped. As Jimmy noted, limited facilities made cooking difficult: “With one stove, we end up standing there waiting forever.” Over time, these conditions reduced his motivation to cook, leading him to rely on simpler alternatives.

Taken together, these examples show how the self-catering system was experienced differently by different prisoners. For many prisoners in well-functioning food groups, the self-catering system enabled the development of a range of food-related skills through shared routines, collaboration, and daily practice. Some prisoners – particularly those outside well-functioning food groups, those with limited cooking skills, or those who viewed food primarily as fuel rather than a social or meaningful practice – expressed a desire to return to the former system of having meals served in the prison dining hall.

#### Support and guidance

While the self-catering system offered potential benefits, some prisoners faced challenges that limited their ability to fully utilise the food-related opportunities and agency it provided. Prisoners described a lack of knowledge, motivation, and practical skills, alongside a need for structured support and guidance. William remarked, “As long as you are strong, it is good, but there are many weak souls here who do not get the help they need.” Similarly, Erik reflected on the burden of managing one’s own diet without adequate support: “But the problem is when it is left up to you to decide (…) the choice was between buying a hamburger or sausages, right, so it wasn’t a healthy diet.”

While some prisoners felt confident in their knowledge about food and nutrition, others struggled to make informed choices. Sebastian, for instance, expressed a strong desire for greater guidance: “I wish I had more knowledge about diet and nutrition. (…) I would like some kind of opportunity, some kind of course.” He emphasised the need for follow-up beyond informal peer support, suggesting that structured programmes could help more prisoners develop healthier eating habits and practical food skills. Many prisoners highlighted the need for individualised support. Erik emphasised the value of having a mentor, comparing it to the role of a teacher or coach who provides encouragement and constructive feedback, particularly when motivation is difficult to sustain.

Together, these narratives show substantial variation in prisoners’ resources for navigating the self-catering system, with several prisoners expressing a need for greater support and guidance.

## Discussion

The four themes identified in this study - food-related agency, social skills and relations, food-related skills, and support and guidance - broadly align with patterns reported in previous research on self-catering systems or prison contexts where prisoners, to some degree, have opportunities to plan, purchase, and prepare their own food. Across these themes, the findings support earlier work suggesting that opportunities for food-related agency are experienced as meaningful and may contribute to a sense of control and normality in everyday prison life (e.g. Groeneveld et al., 2025; Minke & Smoyer, 2017). At the same time, this agency appears to be closely connected to social interactions and relationships. The ways in which shared cooking and eating facilitated relationships, routines, and forms of mutual support, alongside cultural sharing and mutual respect in the present study, are consistent with research emphasising the social, cultural and emotional significance of food practices in prison settings with a relatively high degree of food-related agency (Vanhouche, 2022; Woods-Brown et al., 2023). Furthermore, the development of food-related skills - such as planning, cooking, and budgeting - also reflects patterns identified in previous studies, where self-catering systems can support practical learning, however, in ways that remain uneven and dependent on prisoners’ resources and social environments (Johnson et al., 2024; Minke & Smoyer, 2017; Vanhouche, 2022). Finally, the identified need for support and guidance reinforces a recurring finding in the literature: that not all prisoners are equally positioned to benefit from self-catering arrangements, and that limited skills, knowledge, or resources may constrain the potential benefits of increased agency (Condon et al., 2008; Vanhouche, 2022). Taken together, these findings correspond with prior research in suggesting that while self-catering systems may enable processes associated with health promotion, their effects are contingent and shaped by social, material, and individual conditions.

The prisoners’ experiences illustrate how food-related practices in prison are embedded in broader social and cultural meanings and practices, extending beyond nutritional intake and individual choices. Through everyday food routines such as planning, grocery shopping, cooking, and sharing meals, food practices in the prison context became intertwined with social relationships, identity work, participation and the organisation of daily life, which highlights the multifaceted role of food in the prison environment as outlined by Hervik and Hervik (2025). In line with earlier research, food practices enabled by the self-catering system appeared to promote emotional well-being, strengthen social relationships, support the development of food-related skills and social skills, and contribute to experiences of normality and everyday structure (Minke, 2014; Minke & Smoyer, 2017; Vanhouche, 2022; Woods-Brown et al., 2023). Drawing on Crawford’s (2006) conceptualisation of health as embedded in meaningful everyday practices, these findings suggest that the health-promoting potential of self-catering systems in prison may lie not only in nutritional dimensions, but also in the meaningful forms of participation, identity work, social interaction, and skill development connected to food-related practices in prison. In this way, the findings support a broader understanding of health as something that is produced and negotiated through everyday practices, including those related to food.

The introduction of the self-catering system at Forest Prison was experienced by many prisoners as a meaningful and empowering shift, as it increased their opportunities to make choices, influence everyday routines, and participate actively in social food-related practices. This aligns the present study with previous research suggesting that greater food-related agency may be empowering (Johnson et al., 2024; Woods-Brown et al., 2023). The system enabled practices that prisoners expressed as supporting dimensions of physical, mental and social health and well-being, including strengthening social relationships, promoting healthier eating habits, and enabling identity work. As food represents one of the relatively few areas where prisoners can exercise control, it became a key means through which they could (re)construct and express their identities. For example, some prisoners appeared to move towards new, non-criminal identities through their food choices and food-related practices, such as acquiring cooking skills or adopting healthier diets, consistent with findings reported by Minke (2014).

At the same time, the findings indicate that self-catering is not inherently empowering, but may become empowering depending on social, material, structural and individual conditions. Many of the positive outcomes were reported by prisoners who lived in well-functioning food groups, who had access to supportive relationships, and who possessed personal or practical resources, consistent with earlier research (Minke, 2014; Vanhouche, 2022). In contrast, critical perspectives were more common among those without such support or resources. These individuals faced challenges such as lack of financial resources, social isolation, or limited food-related skills.

There were clear economic differences among prisoners, affecting their ability to manage within the self-catering system, echoing earlier findings where economic constraints have been shown to limit food choices and shape prisoners’ experiences of food-related agency within self-catering systems (Minke & Smoyer, 2017; Vanhouche, 2022). Belonging to a well-functioning food group could partly offset some of these inequalities by allowing prisoners to share costs and resources. These findings echo previous research showing that food groups can be cost-saving (Vanhouche, 2022). In contrast, those without such networks more often experienced greater financial challenges. Furthermore, some prisoners had limited food-related knowledge, skills, and competencies, which may reflect aspects of limited food literacy. However, strong social relationships could support the development of such competencies through learning and influence from fellow prisoners. Overall, the findings suggest that the potential benefits of self-catering were unevenly distributed among prisoners. Limited resources for taking advantage of the relatively high food-related agency the system offered, may therefore represent an important barrier to realising the health-promoting potential of self-catering arrangements. Initiatives such as food education, structured guidance, as well as systematic efforts to establish and support food groups could help reduce these disparities by supporting the development of prisoners’ food literacy and participation within the self-catering system.

Additionally, in the shift to a self-catering system, prisoners reported feeling seen and heard when their protests against the existing food system were acknowledged and acted upon. The change also strengthened their trust in the prison management, illustrating how structural changes that enhance agency can generate positive ripple effects within the prison environment. The transition to a self-catering system may be interpreted as a twofold process of empowerment on a meso and micro level (cf. Kayser et al., 2019): first, prisoners successfully influenced the food system in the direction they preferred; second, the change granted them more control over planning, shopping, and preparing their meals. Both forms of empowerment were seen by participants as beneficial for their health and well-being. Nonetheless, some prisoners would benefit from further empowerment through initiatives such as individualised counselling, cooking skills training, or budget guidance. Without this, inequalities may persist or deepen. Consequently, to realise the potential of self-catering systems for empowerment and health promotion, such systems should be designed and implemented with attention to inequality, support needs, and structural conditions.

Arguably, prison policies that enable self-catering food systems can be understood as empowering at the macro level, with additional potential for empowerment at both the meso and micro levels (cf. Kayser et al., 2019). Consequently, self-catering systems may hold potential for promoting health within prison settings (cf. World Health Organization, 1986) and may serve as one way to counteract the lack of agency that often constrains health promotion in prisons. While the findings are grounded in a specific low-security prison context, the insights from this study may be relevant to other prison settings where self-catering or similar arrangements are implemented or considered for implementation. However, while the findings presented offer important insights, it must be acknowledged that the population in Forest Prison may not reflect the broader prison population. The participants may be more resourceful or better positioned to benefit from self-catering since most of the prisoners in this specific prison have followed the progression of their sentence, complied with institutional rules, and avoided being transferred back to closed prisons. This should be taken into account when considering applicability to other contexts.

## Conclusion

The study illustrates how food-related practices may function as meaningful aspects of everyday life in prison, connected to empowerment, participation, identity work, and social relationships. More broadly, the findings suggest that food systems may shape everyday life and opportunities for health promotion within prison settings. However, prisoners’ opportunities to benefit from self-catering varied considerably depending on factors such as food literacy, social relationships, and access to well-functioning food groups. The findings therefore suggest that the health-promoting potential of self-catering systems lies not only in increased food-related agency itself, but also in the availability of support, guidance, and organisational conditions that enable prisoners to benefit from such arrangements. Overall, the insights from this study may be of relevance for policymakers and prison authorities aiming to facilitate health promotion through food in prisons.

## Data Availability

No datasets were generated or analysed during the current study.
